# Perspectives from deductible plan enrollees: plan knowledge and anticipated care-seeking changes

**DOI:** 10.1186/1472-6963-9-244

**Published:** 2009-12-29

**Authors:** Mary Reed, Nancy Benedetti, Richard Brand, Joseph P Newhouse, John Hsu

**Affiliations:** 1 Center for Health Policy Studies, Division of Research, 2000 Broadway, Oakland, California, 94612, USA; 2 Department of Epidemiology and Biostatistics, University of California at San Francisco, 185 Berry Street, San Francisco, California, 94107, USA; 3 Department of Health Care Policy, Harvard Medical School, 180 Longwood Avenue, Boston, Massachusetts, 02115, USA; 4 Department of Health Policy and Management, Harvard School of Public Health, 677 Huntington Avenue, Boston, Massachusetts, 02115, USA; 5 Harvard Kennedy School of Government, 79 John F Kennedy Street, Cambridge, Massachusetts, 02138, USA; 6 James J Mongan Institute for Health Policy, Massachusetts General Hospital, 50 Staniford Street, Boston, Massachusetts, 02114, USA

## Abstract

**Background:**

Consumer directed health care proposes that patients will engage as informed consumers of health care services by sharing in more of their medical costs, often through deductibles. We examined knowledge of deductible plan details among new enrollees, as well as anticipated care-seeking changes in response to the deductible.

**Methods:**

In a large integrated delivery system with a range of deductible-based health plans which varied in services included or exempted from deductible, we conducted a mixed-method, cross-sectional telephone interview study.

**Results:**

Among 458 adults newly enrolled in a deductible plan (71% response rate), 51% knew they had a deductible, 26% knew the deductible amount, and 6% knew which medical services were included or exempted from their deductible. After adjusting for respondent characteristics, those with more deductible-applicable services and those with lower self-reported health status were significantly more likely to know they had a deductible. Among those who knew of their deductible, half anticipated that it would cause them to delay or avoid medical care, including avoiding doctor's office visits and medical tests, even services that they believed were medically necessary. Many expressed concern about their costs, anticipating the inability to afford care and expressing the desire to change plans.

**Conclusion:**

Early in their experience with a deductible, patients had limited awareness of the deductible and little knowledge of the details. Many who knew of the deductible reported that it would cause them to delay or avoid seeking care and were concerned about their healthcare costs.

## Background

An increasing number of patients are facing higher health insurance deductibles [1]. Often, preventive medical services are exempted from the deductible, which removes the cost barrier for those services, but adds to the complexity of the plan. While proponents suggest that having "skin in the game," helps patients avoid unnecessary care, some have raised concerns about the role of patients as informed consumers and questioned plans' effects on medical services utilization [1-7].

For the incentives to work, it is important for patients to understand their cost-sharing plan and be able to anticipate how much they will have to pay for various services when they need them. Currently, little is known about how well patients understand their deductible plans, and how they adapt their care-seeking behavior under these plans [8,9]. Since deductible plans have been shown to reduce patients' use of health services [10,11], it is important to examine whether new deductible plan enrollees plan to change their care-seeking behavior in response to the deductible.

In this study we examine deductible plan enrollees' knowledge of their plan at the beginning of the coverage year, and describe qualitative reports of anticipated changes in care-seeking in response to the deductible.

## Methods

### Setting/Population

Kaiser Permanente Northern California (KPNC) is an integrated delivery system (IDS) which newly began offering deductible health plans to enrollees of large employer groups in 2005. Cost-sharing plans varied by employer, but enrollees could not self-select their KPNC cost-sharing level. All study respondents had one of two types of deductible plans: the more generous plan had a deductible for hospital care and emergency department (ED) visits only; the less generous plan additionally included medical tests in the deductible. In both plans, patients paid a copayment for all office visits and generic drugs; some also faced a separate drug deductible for brand-name drugs only. Family plan deductible amounts were twice the individual deductible amount.

Our source population included all adult (age ≥18) health plan members enrolled in a deductible plan through a large-group employer in January 2005 who had also been members in January 2004. We obtained a stratified random sample of 1,100 members: 550 from the population without a chronic disease and 550 from patients in the health plan's chronic disease registries for asthma, hypertension or diabetes. Since we did not find a statistically significant difference between the chronic and non-chronic disease populations in any of the analyses for this paper, we pooled these two groups. Telephone study eligibility criteria excluded those who did not have valid contact information (n = 139), were unreachable (n = 187), or with language barriers, cognitive impairment, or severe illness (n = 47); we also excluded those who died (n = 1) or left the health system or deductible plan (n = 72).

### Data

In early 2005, we mailed a study introduction letter with a pre-paid reply postcard to our study sample. Research assistants called all who did not refuse participation (up to 15 times during various times of the day and week) obtaining verbal consent before beginning the interview. The Kaiser Foundation Research Institute Institutional Review Board approved the study protocol. Overall, 71% (n = 458) of all eligible subjects participated in the study: 74% from the chronic disease sample and 67% from the non-chronic disease population. Respondents were similar to non-respondents with respect to age, gender, plan characteristics, and chronic disease status (p > 0.05).

To assess deductible knowledge, we asked if respondents had any medical deductible, the deductible amount, and if they had to pay the full price before reaching their deductible for hospital care, ED visits, medical tests, preventive office visits or non-preventive office visits. If respondents were unsure, the interviewer encouraged them to make an educated guess. For respondents who reported a deductible, we asked an open-ended question about how it would affect their care-seeking ("How do you think having a deductible will affect your choices and behavior regarding your healthcare?").

We also collected respondents' race/ethnicity, education level, marital status, annual household income, and health status. We obtained information on respondents' age, gender, and actual cost-sharing from health system administrative databases.

### Analysis

To assess cost-sharing knowledge, we calculated the percent of respondents who correctly reported having a deductible, and which services were included in the deductible. We considered the respondent correct if their self-reported deductible matched either their individual or family deductible amount. Since the study over-sampled patients with chronic disease, all analyses were either stratified or weighted. All multivariate analyses also adjust for this stratification variable. To examine the association between deductible knowledge and respondent characteristics, we used multiple logistic regression. We included the missing responses as a separate category for each variable.

We coded respondents' open-ended descriptions of the anticipated effect of their deductible on care-seeking into 13 specific categories, with responses coded into as many categories as applied (from one to four categories per respondent). We then condensed these categories into four general themes (Table 1), grouping reports of changes in care-seeking into 'Decrease any medical care', reports of concerns or dissatisfaction with health care costs into 'Concern about costs', reports that the deductible would not affect care-seeking into 'No Change' and reports that of uncertain effects on care-seeking into 'Don't know/Not Sure'. Three researchers coded responses independently, with disagreements in coding resolved by assigning the code used by a majority of coders.

**Table 1:** Characteristics of Respondents

	Total	Non-Chronic Disease	Chronic Disease
Female gender*	55.8%	57.7%	48.4%
Age*:			
18-29 years	16.1%	18.3%	7.6%
30-49 years	54.6%	59.1%	37.2%
50-64 years	26.7%	21.2%	48.0%
65+ years	2.6%	1.4%	7.2%
Race: Non-white	36.4%	38.0%	30.7%
Married	65.8%	65.8%	65.9%
Education: Less than college graduate	68.1%	68.8%	65.5%
Household income: <$35,000	16.0%	15.2%	19.0%
Self-reported health: Excellent or very good*	56.1%	60.1%	40.8%

*p < 0.05 between samples.

Total percentages are population estimates weighted for sampling proportions.

## Results

Participants had a mean age of 44.8 years, 56% were female, 76.0% reported white race/ethnicity, 44% had "excellent" or "very good" health, 68% had not graduated from college, and 16% had incomes of < $35,000 (Table 1). Individual medical deductible amounts were $250-$1000 for the more generous plan and $500-$1000 for the less generous plan; 66% of respondents had a family plan; 46% had a brand-name drug deductible.

### Knowledge

**Figure 1:**
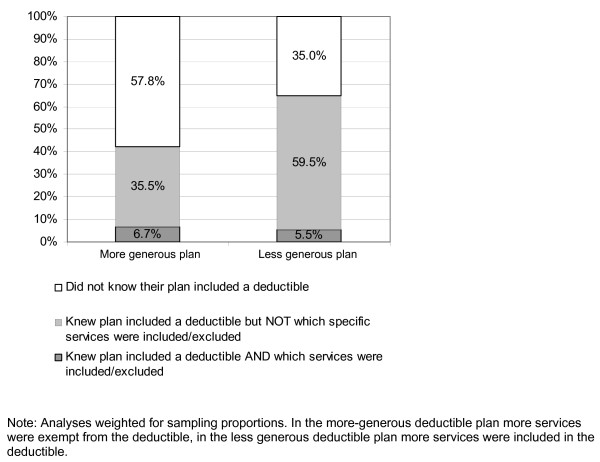
**Respondent knowledge of having a deductible and of deductible-covered services**.

Overall, 49% of all respondents did not know that their plan included a deductible in 2005 (57.8% in the more generous plan, 35.0% in the less generous plan). Of all respondents, 26% did know their deductible amount and 6% knew which specific services were included or excluded from the deductible. Altogether, 5% of all respondents correctly knew that they had a deductible, knew the deductible amount and knew which services applied to the deductible. Figure 1 shows respondents' knowledge of having a deductible, and deductible covered services by plan generosity.

While hospital care and emergency department visits were included in all respondents' deductibles, 81% did not know that they had to pay the full price before reaching the deductible. Among patients' whose deductible included medical tests (less generous plan), 72% did not know that they had to pay the full price; among those whose deductible excluded medical tests (more generous plan), 38% mistakenly thought that they had to pay the full price for medical tests. Office visits were excluded from all deductibles in this population, but 11% of all respondents mistakenly thought that they had to pay the full price for office visits.

In multivariate analyses (see Additional File 1), respondents with a less generous plan and those with a separate prescription drug deductible were significantly more likely to know they had a medical deductible, and respondents with excellent/very good self-reported health status were less likely to know they had a deductible.

### Anticipated changes in care-seeking

Among respondents who knew of their deductible, 83% responded to the open-ended question about its affect on their care-seeking behavior. We coded all responses into four broad themes with 13 sub-categories (allowing for coding into multiple categories). Table 2 shows each of the specific response categories and the general themes, the percent of respondents whose response fit into these categories and a brief quotation as a key example of those responses.

**Table 2:** Anticipated changes in care-seeking: "How do you think having a deductible will affect your choices and behavior regarding your health care?

Care-seeking behavior change category	%	*Key Example*
Decrease or delay medical care	50.8%	
Use less care (type not specified)	26.1%	*I know it's going to cost quite a bit, so I won't go quite as much*.
Go to the doctor less	9.6%	*I don't make any doctor appointments, and don't go to my made appointments. I need to go to a specialist. I will probably spend at least $500 a year [deductible amount]*.
Get fewer medical tests	4.4%	*It's going to lower my ability to have healthcare because I can't afford it. I have 6 different chronic diseases and have to put off blood tests for 3 months. This means I can't afford to get better - it's too expensive to get healthy now*.
Go the ED less	3.1%	*I'd just wait until I was sure it was dangerous before shelling out all that money for nothing*.
Use fewer prescription drugs	2.2%	*It affects a lot -This year I'm trying not to refill the new expensive medication*.
Reconsider/Delay seeking medical care	11.2%	*I will re-think decisions up to the deductible. It's pricey with three kids*.
Use out-of system care	2.4%	*I will go to a free clinic instead*.
		
Concerned about deductible costs	25.8%	
Change to another health plan	15.1%	*I have looked for another insurance carrier, and as soon as I find one we will change*.
Can't afford health care	14.7%	*I can't afford to take all medications I need. I might even have to buy less food to afford medication. I am going to look into other plans*.
Unhappy with my deductible plan	8.3%	*This plan wasn't a good choice, it's easier to have a flat rate instead of a guessing game*.
Uncertain about my costs	6.5%	*We pay so much money and I don't know how much anything costs*.
		
Uncertain about effects	3.8%	*I really don't know - I'm very healthy*.
No expected effects	24.0%	*It doesn't impact me at all. I should help pay for my medical services, so it's pretty fair*.

Question asked among respondents who knew they faced a deductible (n = 172). Respondents could report responses in multiple categories. Percentages are weighted for sampling proportions.

Overall, 51% of respondents reported that the deductible would cause them to decrease or delay their use of medical care, including going to the doctor less, getting fewer medical tests, going to the emergency room less, and thinking twice about or delaying care. Often these respondents described that they would reduce care-seeking even for types of care that they believed were clinically necessary (see examples in Table 2). In addition, 26% of respondents indicated that they were concerned about their costs under the deductible plan, including wanting to change to another health plan, having difficulty affording health care, being unhappy with the deductible plan, and being uncertain about health care costs under this plan (see examples in Table 2). Many respondents also reported that they did not expect to change their care-seeking behavior (24%). A small percent of respondents reported that they were uncertain about how the deductible would affect their care-seeking behavior (4%).

## Discussion

Early in their experience with a new deductible plan, we found that only half of respondents knew they had a deductible and only a quarter knew the amount. Very few knew which specific services were included in their deductible. Over half of those who knew of their deductible reported that they planned to delay or avoid seeking medical care in response.

We found that respondents in the less generous plan were significantly more likely to know they faced a medical deductible, as were those with a prescription drug deductible. Those with worse self-reported health were significantly more likely to know of their deductible, suggesting that patients with more clinical need or use may be more knowledgeable about their health plan, but in multivariate analyses we did not find significant differences in plan knowledge between patients with and without chronic diseases. Particularly among patients with more clinical need, patient knowledge of benefits would likely improve somewhat with longer-term experience with these plans. Still, we found that exceedingly few respondents (6%) knew exactly which services were included in or exempt from their deductible, which raises concerns about patients' ability to successfully navigate the health-care system while facing the complex benefit structures of most deductible plans. For example, patients who are unaware of plan exemptions may unnecessarily avoid preventive care. Our previous studies showed similarly that patients have limited knowledge of their cost-sharing details in non-deductible plans [8,9]. Employers and health plans need to be especially proactive in educating patients about the details of their plans during transitions in complex deductible benefit structures.

Almost half of respondents reported that the deductible would cause them to decrease their use of health care. One in ten reported the deductible would cause them to reduce physician visits, even though office visits were excluded from the deductible. Over 25 percent reported having concerns about costs under the deductible. Still, almost one out of four respondents did report that deductible costs would not affect their care-seeking behavior. Despite frequent reports of planned changes in care-seeking, patients' actual care-seeking decisions may differ. It is important to continue monitoring the ongoing effects of deductibles and other consumer directed health plans on patient care-seeking behavior to understand which types of patients change their behavior, and whether patients respond by reducing discretionary services or avoiding clinically urgent medical care.

While some early studies of deductible plan configurations did not find that patients were deterred form seeking preventive-care [2,12], patient vulnerability to out-of-pocket costs varies widely according to the features in their plan design, including health savings accounts or reimbursement arrangements (HSA or HRA). It is also important to study patient knowledge and behavior across these plan variations. While the deductibles in our study were relatively modest for so-called high deductible plans, the plans were not supplemented by an employer contribution to an HSA or HRA. Similarly, while the study findings are based on experiences within one integrated delivery system, patients elsewhere with higher deductible levels could make even larger changes in care-seeking and be more concerned about these out-of-pocket costs.

## Conclusions

Patients newly enrolled in a deductible plan had limited knowledge of having any deductible, including rarely being aware of which services are deductible-exempt. The limited knowledge raises concerns about patients' ability to engage as informed consumers of healthcare, especially in an environment of consumer-directed health plan designs with additional complex features such as healthcare savings accounts. At minimum, more effective efforts to educate consumers about the working of their plan are needed.

Half the patients who knew of their deductible reported that they planned to delay or avoid care. It is important to continue to monitor patient perceptions of their benefit plan and to determine if poor knowledge of deductible health plans combined with decreased health care utilization adversely affects health care outcomes.

## Competing interests

Newhouse is a director of and holds equity in Aetna, which sells high deductible health plans. The other authors declare that they have no competing interests

## Authors' contributions

MR made substantial contributions to the study's conception and design; data collection, analysis and interpretation; drafted and revised the manuscript critically for important intellectual content. NB made substantial contributions to the analysis and interpretation of the data, and revised the manuscript critically for important intellectual content. RB made substantial contributions to the study's design, analysis and interpretation of the data, and revised the manuscript critically for important intellectual content. JPN made substantial contributions to the study's design, analysis and interpretation of the data, and revised the manuscript critically for important intellectual content. JH made substantial contributions to the study's conception and design; data collection, analysis and interpretation; and revised the manuscript critically for important intellectual content. All authors read and approved the final manuscript.

## Pre-publication history

The pre-publication history for this paper can be accessed here:

http://www.biomedcentral.com/1472-6963/9/244/prepub

## Supplementary Material

Additional file 1 **Respondent characteristics associated with knowledge of having a medical deductible**. Table displays the adjusted odds ratio for correctly reporting having a medical deductible or prescription drug deductible from multiple logistic regression weighted for sampling proportions. Click here for file
