# Supplementary material for: Perspectives from deductible plan enrollees: plan knowledge and anticipated care-seeking changes
Source: BMC Health Serv Res. 2009 Dec 29;9:244. doi: 10.1186/1472-6963-9-244 (PMC2811111; doi:10.1186/1472-6963-9-244)
Supplement: Additional file 1 — Respondent characteristics associated with knowledge of having a medical deductible. Table displays the adjusted odds ratio for correctly reporting having a medical deductible or prescription drug deductible from multiple logistic regression weighted for sampling proportions. [file 1472-6963-9-244-S1.DOC]

| Participant Characteristics | Odds Ratio | 95% | CI |
| --- | --- | --- | --- |
| Plan-type: Less generous vs More generous | ^†^1.91 | 1.11, | 3.29 |
| Having a drug deductible | ^†^1.88 | 1.07, | 3.30 |
| Chronic disease sample | 1.44 | 0.93, | 2.23 |
| Age: 65+ | 0.70 | 0.25, | 1.96 |
| Female | 1.57 | 0.95, | 2.60 |
| Education: Less than college graduate | 0.99 | 0.58, | 1.71 |
| Income: <$35,000 | 1.05 | 0.50, | 2.21 |
| Self-reported health status: Excellent or very good | 0.49 | 0.29, | 0.82 |
| Race: Non-white | 0.75 | 0.44, | 1.27 |
| Married | 1.29 | 0.74, | 2.23 |

^†^p-value<0.05

Table displays the adjusted odds ratio for correctly reporting having a medical deductible or prescription drug deductible from multiple logistic regression weighted for sampling proportions.
